# Basic Pathological Mechanisms in Peripheral Nerve Diseases

**DOI:** 10.3390/ijms26073377

**Published:** 2025-04-04

**Authors:** Angelo Schenone, Sara Massucco, Cristina Schenone, Consuelo Barbara Venturi, Paolo Nozza, Valeria Prada, Tania Pomili, Irene Di Patrizi, Giovanna Capodivento, Lucilla Nobbio, Marina Grandis

**Affiliations:** 1 Department of Neurosciences, Rehabilitation, Ophthalmology, Genetic and Maternal and Infantile Sciences (DINOGMI), University of Genoa, Largo P. Daneo 3, 16132 Genova, Italy; aschenone@neurologia.unige.it (A.S.); crischenone92@gmail.com (C.S.); marina.grandis@unige.it (M.G.); 2 IRCCS Ospedale Policlinico San Martino, UO Clinica Neurologica, Largo R. Benzi 10, 16132 Genova, Italy; giovanna.capodivento@hsanmartino.it (G.C.); lucilla.nobbio@hsanmartino.it (L.N.); 3 IRCCS Ospedale Policlinico San Martino, UO Patologia, Largo R. Benzi 10, 16132 Genova, Italy; consuelobarbara.venturi@hsanmartino.it (C.B.V.); paolo.nozza@hsanmartino.it (P.N.); 4 Italian Multiple Sclerosis Foundation (FISM), Scientific Research Area, Via Operai 40, 16149 Genoa, Italy; valeria.prada@aism.it; 5 Electron Microscopy Facility, Istituto Italiano di Tecnologia, Via Morego 30, 16163 Genova, Italy; tania.pomili@iit.it; 6 IRCCS Ospedale Policlinico San Martino, UO Radiologia, Largo R. Benzi 10, 16132 Genova, Italy; irene.dipatrizi@gmail.com

**Keywords:** peripheral nervous system, myelin, axon, neuropathies, nerve

## Abstract

Pathological changes and the cellular and molecular mechanisms underlying axonopathy and myelinopathy are key to understanding a wide range of inherited and acquired peripheral nerve disorders. While the clinical indications for nerve biopsy have diminished over time, its diagnostic value remains significant in select conditions, offering a unique window into the pathophysiological processes of peripheral neuropathies. Evidence highlights the symbiotic relationship between axons and myelinating Schwann cells, wherein disruptions in axo-glial interactions contribute to neuropathogenesis. This review synthesizes recent insights into the pathological and molecular underpinnings of axonopathy and myelinopathy. Axonopathy encompasses Wallerian degeneration, axonal atrophy, and dystrophy. Although extensively studied in traumatic nerve injury, the mechanisms of axonal degeneration and Schwann cell-mediated repair are increasingly recognized as pivotal in non-traumatic disorders, including dying-back neuropathies. We briefly outline key transcription factors, signaling pathways, and epigenetic changes driving axonal regeneration. For myelinopathy, we discuss primary segmental demyelination and dysmyelination, characterized by defective myelin development. We describe paranodal demyelination in light of recent findings in nodopathies, emphasizing that it is not an exclusive indicator of demyelinating disorders. This comprehensive review provides a framework to enhance our understanding of peripheral nerve pathology and its implications for developing targeted therapies.

## 1. Introduction

Peripheral neuropathies have a prevalence of 2.4% in the general population, increasing to 8% in individuals over the age of 55, making them one of the most common neurological disorders [1]. There are hundreds of potential causes of peripheral neuropathy [2]. Diagnosis is typically established based on medical history, physical examination, nerve conduction studies, laboratory tests, and, in some cases, imaging, genetic testing, and cerebrospinal fluid analysis [3]. As an invasive procedure that results in a localized area of cutaneous anesthesia on the lateral aspect of the foot, sural nerve biopsy represents the final step in the diagnostic workup of peripheral neuropathies [3]. Over the years, advancements in genetics, immunology, and imaging have significantly reduced its indications [3,4]. Nonetheless, sural nerve biopsy remains essential in certain cases, as it can help identify treatable causes of neuropathy, such as vasculitis isolated to the peripheral nervous system (PNS). In a study of over a thousand sural nerve biopsies, the procedure led to a definitive diagnosis in more than half of the cases, while 34% of the reports were inconclusive, particularly when there was no clear clinical suspicion prior to the biopsy [3]. The primary indication for nerve biopsy is the suspicion of interstitial neuropathies, such as vasculitis, granulomatosis, amyloidosis, or tumors [3,5,6]. Moreover, neuropathological studies play a crucial role in deepening our understanding of the pathophysiological mechanisms underlying peripheral neuropathies [7], shedding light on nerve damage progression, molecular pathways, and potential therapeutic targets.

PNS lesions can be classified into two major basic patterns based on their neuropathological characteristics: (a) axonopathy, which includes axonal (Wallerian) degeneration (WD), axonal atrophy, and axonal dystrophy, and (b) myelinopathy, which consists mainly of primary segmental demyelination and dysmyelination. These patterns often coexist and can occur simultaneously or sequentially, such as in sublethal degenerative axonal changes or axonal atrophy resulting in secondary demyelination. Moreover, damage to the cell body (neuronopathy) can serve as an early and critical target, inducing secondary changes in the peripheral nerves.

In this review, we will describe the key mechanisms underlying peripheral nerve damage, providing a comprehensive discussion of the pathological and molecular changes at the core of major peripheral neuropathies.

## 2. Axonopathy

### 2.1. Active Axonal (Wallerian) Degeneration

**Figure 1:**
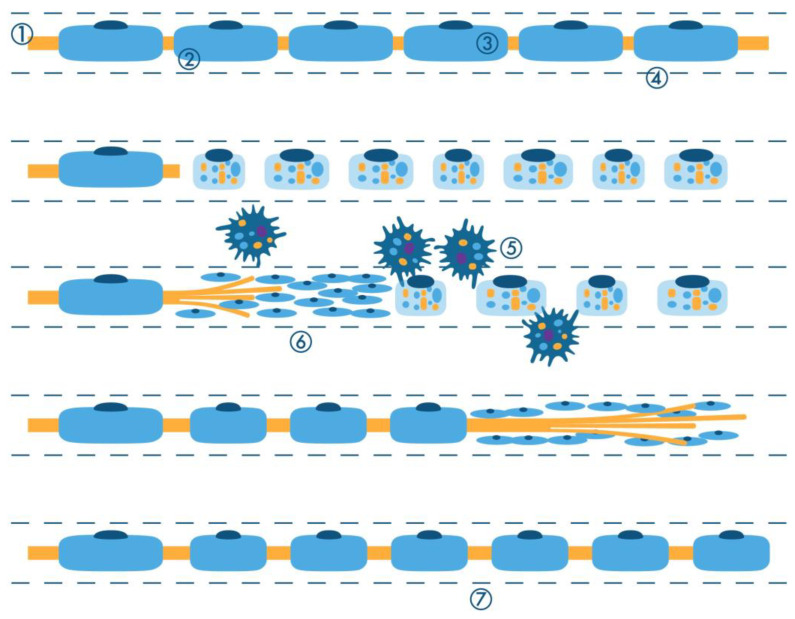
Graphical representation of axonal (Wallerian) degeneration. 1 = axon; 2 = site of axonal crush; 3 = Schwann cell; 4 = basal lamina; 5 = macrophages phagocytizing myelin and axonal debris; 6 = proliferating Schwann cells; 7 = regeneration and remyelination result in internodal distances distal to the injury that are uniformly shorter compared to those formed during developmental myelination.

As described by Waller and Cajal in the nineteenth and early twentieth centuries [8,9], this term refers to the degenerative phenomena of the axon and myelin that develop distally to the injury site (Figure 1). The crush and transection of axons are the most common and deeply studied mechanisms of nerve damage leading to axonal degeneration [9,10]. Additional mechanisms may encompass toxins, including pharmaceutical agents, and vascular lesions inducing ischemic damage to the nerves, such as vasculitis. After an axonal injury, a cascade of degradation processes ensues, manifesting in molecular and morphological changes within the axons, Schwann cells (SCs), and macrophages. Despite this, the conduction of impulses can persist for as long as 48 h distal to the site of axonal transection [11], and electromyography only reveals signs of denervation after a period of 10 to 15 days from the initial injury [12].

#### 2.1.1. Changes in the Axon

**Figure 2:**
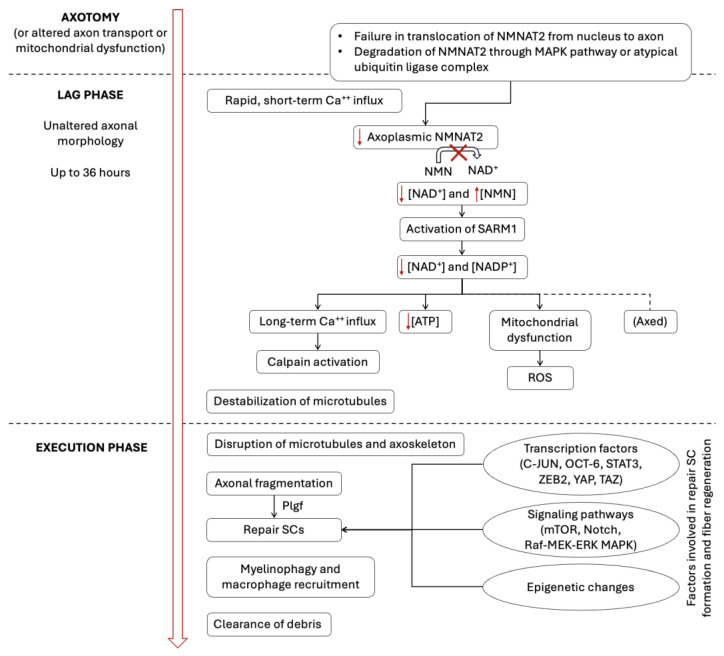
Molecular pathways involved in axonal degeneration and regeneration. Following axotomy, the axon separated from its soma enters a lag phase, during which its morphology remains intact while several subcellular processes unfold, ultimately resulting in energy deficits, ROS production, and calpain activation, leading to the execution phase. NMNAT2 plays a critical role in axon survival and is continuously transported from the nucleus to the axon; thus, axotomy and impaired axonal transport result in a drop in axoplasmic NMNAT2. The MAPK pathway and the atypical ubiquitin ligase complex drive the degradation of vesicle-associated and soluble NMNAT2, respectively. NMNAT2 sustains constant NAD^+^ levels and low NMN concentration. A reduction in axoplasmic NMNAT2 leads to decreased NAD^+^ and elevated NMN concentrations, triggering SARM1 activation. SARM1 activation depletes NAD^+^ and NADP^+^, resulting in ATP depletion, mitochondrial dysfunction with ROS production, and Ca^++^ influx, culminating in calpain activation. Increased Axed levels have also been described in Drosophila models. During the execution phase, axon fragmentation occurs. Plgf released from the damaged axon facilitates the constriction of actin filaments in SCs, promoting axon fragmentation. SCs then undergo both biochemical and morphological changes, transitioning into repair SCs. The formation of repair SCs and subsequent fiber regeneration are driven by multiple transcription factors, signaling pathways, and epigenetic modifications, some of which are illustrated in the figure and described in Section 2.1.3. ATP = adenosine triphosphate; MAPK = mitogen-activated protein kinase; NAD^+^ = nicotinamide adenine dinucleotide; NADP^+^ = nicotinamide adenine dinucleotide phosphate; NMN = nicotinamide mononucleotide; NMNAT2 = nicotinamide mononucleotide adenylyltransferase 2; Plgf = placental growth factor; ROS = reactive oxygen species; SARM1 = sterile alpha/Armadillo/Toll-Interleukin receptor homology domain 1 protein; SC = Schwann cell.

Subsequent to axotomy, the axon that has been separated from the soma goes through a lag phase, during which its overall morphology remains unchanged for 6–24 h [13]. In this “molecular phase”, calcium (Ca^++^) levels in the axon increase rapidly and transiently within hours of injury. This increase is observed in both the proximal and distal parts of the axon. This increase in Ca^++^ influx is attributed to the opening of ion-specific channels and, to a lesser extent, to the release from axon internal Ca^++^ stores, leading to the accumulation of free intracellular Ca^++^ [13,14,15,16,17]. After the initial, rapid, brief Ca^++^ surge, a sequence of additional molecular events ensues, encompassing the depletion of nicotinamide adenine dinucleotide (NAD^+^) and adenosine triphosphate [18,19,20] and the activation of calpains (Figure 2). An impairment of axonal energy homeostasis occurs, leading to the “execution phase” of axonal degeneration [13]. This phase is characterized by a massive decrease in microtubular and neurofilament protein levels [21,22]; the presence of watery, unstructured axoplasm; and subsequent axonal fragmentation [23]. At this juncture, the sequence of active axonal degeneration has begun, with prompt degradation of the axoplasm, axolemma, and myelin sheath distal to the site of the nerve lesion. Axonal fragmentation and the degradation of neurofilament proteins are in progress before the occurrence of the significant recruitment of macrophages and SC proliferation, which finally lead to the clearance of the distal stump of axonal and myelin debris [23]. These changes may occur even more rapidly in small axons, which SCs and macrophages absorb [24].

Axonal degeneration is not merely a passive process resulting from the separation of the axonal fragment from the cell body; rather, it is an active process that occurs independently of classic apoptotic cascades [25]. Several specific enzymatic reactions regulate the progression of degeneration, as well as the maintenance of the structural integrity of healthy axons.

The active nature of WD is most typically demonstrated by the identification of naturally occurring mutant mice, the Wld^s^ (WD slow) mice, in which the progression of WD is delayed for up to three weeks [26]. In these mice, the delayed degeneration phenotype is inherited as a dominant trait, suggesting that genetic factors are involved in the normal progression of WD [23]. The genetic change that causes the Wld^s^ phenotype was identified as a triplication of an approximately 85-kilobase region on mouse chromosome 4 [27,28,29]. Recent studies have suggested that, in addition to its role in delaying WD, the genetic alteration in Wld^s^ mice exerts an influence on neuronal development and physiological processes, including neural circuit formation and ocular dominance plasticity [30,31,32,33]. The increased expression of the Wld fusion protein results in a delay in axonal degeneration. This is attributable to the molecular configuration of the Wld protein, which consists of an N-terminal 70-amino acid fragment of the ubiquitin ligase UBE4B (ubiquitin conjugation factor E4B) devoid of catalytic activity and the full-length nicotinamide mononucleotide adenylyltransferase 1 (NMNAT1), which catalyzes NAD^+^ synthesis [29]. The NMNAT enzymatic activity has been identified as a contributing factor to the delayed degeneration phenotype [34]. The translocation of NMNAT from the nucleus to the axon in neurons, along with its increased activity, results in the activation of Sirt1, a member of the sirtuin family of NAD^+^-dependent deacetylases [35]. Notably, mice with the forced expression of distinct forms of NMNAT also exhibit the delayed axonal degeneration phenotype, which encompasses mice overexpressing cytoplasmic mutant forms of NMNAT1, NMNAT2, and NMNAT3, but not wild-type NMNAT1 [35]. It is now well established that increased activity of the NAD^+^-dependent deacetylase Sirt1 also protects neurons and axons from degeneration, as may happen in other conditions like experimental toxic neuropathies or even Alzheimer’s disease [35]. The significance of the NAD^+^ pathway in regulating axonal degeneration is strengthened by the observation that the degradation of axonal NAD^+^ induced by the Drosophila sterile a/Armadillo/Toll-Interleukin receptor homology domain (Sarm) protein results in Sarm1-induced axonal degeneration [35,36]. Accordingly, Sarm1 knockout mice exhibit a significant delay in the progression of WD, akin to the observed delay in Wld^s^ mice [37].

Several additional signaling pathways are involved in regulating axonal degeneration subsequent to axonal damage (for a review, see Rosell A. and Neukomm L.J., 2019 [13]). These include the elevation of reactive oxygen species (ROS), the activation of the ubiquitin ligase zinc and ring finger 1, which is a downstream event of ROS-induced epidermal growth factor receptor-dependent phosphorylation, and the dual leucine kinase which promotes the degeneration of severed axons in Drosophila and mice through its target c-Jun N-terminal kinases (JNKs) [38,39]. The phosphorylation of JNKs is also triggered by the activation of the mitogen-activated protein kinase (MAPK) signaling pathway, which occurs within five minutes after axonal injury and, in turn, downregulates the expression of the axon-protecting NMNAT2 protein [13,40]. Recently, an additional critical mediator of axon degeneration signaling has been identified in Drosophila: the axed gene, which consists of two isoforms (axed^long^ and axed^short^). These isoforms code for axed proteins, which are predominantly found in axons and synapses. The levels of these proteins increase 4–6 h post axotomy and return to baseline 24 h after injury [41]. Most of these findings arise from experimental models employing rodents, namely, in vitro cultured neurons from superior cell ganglia and dorsal root ganglia from the PNS, along with in vivo sciatic nerve lesions.

In the longest axons, the distal portion may be preferentially affected, a phenomenon that has been observed in numerous experimental and human chronic neuropathies. These include diabetic neuropathy, toxic neuropathies resulting from chemotherapy, alcohol, or isoniazid, and neuropathies resulting from viral infections such as human immunodeficiency virus (HIV) or hepatitis C virus (HCV). In these conditions, axonal degeneration appears to spread from the distal axonal segment towards the cell body, a phenomenon referred to as “**dying-back axonal degeneration**”. This process shares molecular pathways with WD [42,43,44]. For instance, a deficiency of NMNAT2 (see above) due to axonal transport block or other conditions, such as increased proteasome activity, can induce axonal degeneration [45]. This observation leads to the concept that the distal axon is at the greatest risk of degeneration because of its distance from the synthetic source of the neuron [24]. Axonal transport consists of the bidirectional trafficking of various organelles and cargoes. Axonal transport can be fast or slow: vesicular cargoes move relatively quickly (50–400 mm/day), whereas the transport of soluble (cytosolic) and cytoskeletal proteins takes much longer (less than 8 mm/day) [46]. Microtubules (MTs) are an important structural component of axonal transport. Due to the uniform orientation of axons, kinesins and dyneins are in charge of the anterograde (from soma to tips) and retrograde (from tips to soma) transport of proteins and organelles, respectively [47]. Damage to MTs may be responsible for a block of axonal transport, leading to dying-back neuropathy, as occurs in chemotherapy-induced peripheral neuropathies [45]. The most common causes of chemotherapy-induced neuropathy are platinum-based compounds, anti-tubulin drugs, and proteasome inhibitors. These agents share a common pathway that triggers early axonal degeneration, which may be mediated mainly by their negative effect on the MT system [45].

Finally, another mechanism of dying-back neuropathy is the accumulation of mitochondrial pathology due to oxidative stress to the mitochondrial genome or problems in mitochondrial calcium homeostasis, which may lead to distal axonopathy [24].

**Figure 3:**
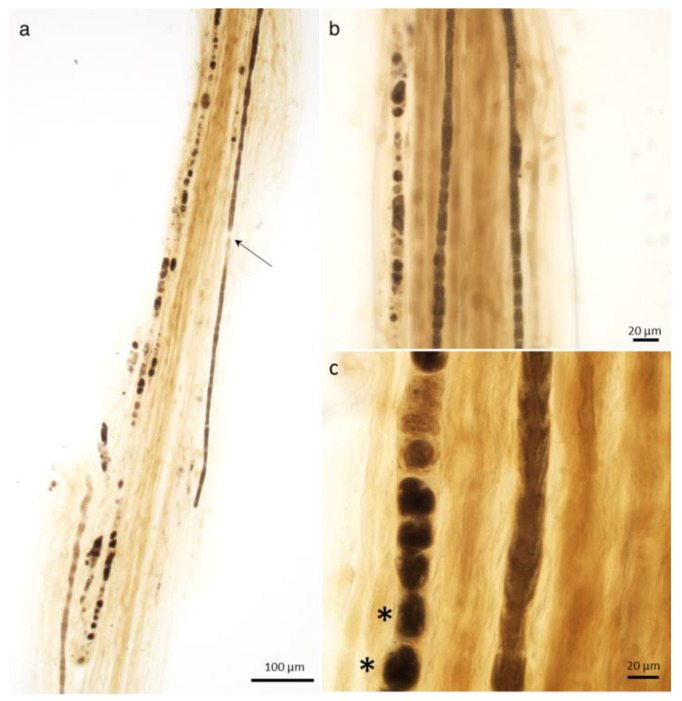
Axonopathy. Wallerian degeneration. Initially, there is retraction of the paranodal myelin ((**a**), arrow) and widening of the Schmidt–Lanterman clefts, where myelin degradation into short segments (“myelin ovoids”, asterisks) begins. Teased nerve fiber preparation, (**a**) 10×; (**b**) 40×; (**c**) 100× oil, * = myelin ovoids.

After an axonal injury, the myelin sheath undergoes catabolic changes and is segmented into ovoids that become progressively smaller and more separated from each other, and the axon becomes thinner and fragmented until it disappears. Initially, there is retraction of the paranodal myelin and widening of the Schmidt–Lanterman clefts, where myelin degradation into short segments (“myelin ovoids”) begins [48,49] (Figure 3).

Three days after nerve injury, SCs proliferate and recruit macrophages to phagocytize debris from degenerated axons and myelin sheaths. Macrophages reach peak density one week after the injury and, in combination with the action of SCs, support the reinnervation process by promoting angiogenesis and regeneration [50].

#### 2.1.2. Schwann Cell and Macrophage Activity

Resident myelinating and nonmyelinating SCs play major roles in both the degeneration and regeneration phases of nerve injury. Positive signals from the injured axon, such as placental growth factor signaling [51], trigger SCs to start the initial destruction and early degradation of myelin and axons [52,53]. In the very first stages of axonal degeneration, SCs begin to accumulate axonal and myelin debris in their cytoplasm and downregulate the expression of myelin-related proteins, such as myelin protein zero (MPZ) and peripheral myelin protein of 22 kilodaltons (PMP22) [44,53].

To ingest their own myelin sheaths, SCs use a form of macroautophagy called myelinophagy [54]. Lipidated LC3, a marker of autophagosomes, is highly expressed in addition to many autophagy machinery genes, such as autophagy-related gene 7 (*Atg7*) [51]. In addition, the molecular phenotype of SCs shifts to resemble that of premyelinating or nonmyelinating SCs. Some molecules such as the low-affinity NGF receptor p75, glial fibrillary protein, n-cadherin, glial maturation factor β, cell adhesion molecules L1, nerve cell adhesion molecule (NCAM), Pax3 (a myelin gene repressor), and c-Jun are re-expressed, whereas the transcription factor Krox-2 is downregulated [44,55,56,57].

Additional molecular activities of the repair program include the expression of various cytokines and chemokines and the upregulation of genes and proteins that are important for promoting axon guidance and neuronal survival, such as trophic factors, including glial cell-derived neurotrophic factor, brain-derived neurotrophic factor, neurotrophin-3, sonic hedgehog, semaphorins, and ephrins, in addition to cell adhesion and matrix molecules such as integrins, collagens, and matrix metalloproteins [51]. These molecular and biochemical changes combine to induce the proliferation of SCs and their morphological transformation into repair SCs [51]. Mitotically quiescent SCs in the distal stump begin to proliferate, adopt a longer, bipolar, branched morphology, and aggregate with neighboring cells within their basal lamina tubes, forming the bands of Büngner [58]. The correct formation of the bands of Büngner likely underlies efficient axon regeneration [59]. SC proliferation is greater in nerves with large, myelinated axons than in nerves with predominantly small fibers [24]. These changes involve cell-to-cell communication between the axonal growth cone and SCs. Three to seven days after axotomy, SCs upregulate the expression of neuregulin (NRG-1) type I, which, in turn, affects the expression of membrane-bound NRG-1 type III on axons. Axonal NRG-1 and the erbB receptor complex expressed on SCs appear to be largely dispensable for demyelination and repair SC formation [60,61,62]. At the same time, there is an upregulation of the SC erbB2 and erbB3 neuregulin receptors. Axonal NRG-1 and SC erbB signaling influence the rate of axonal regeneration, and both axonal NRG-1 type III and SC-produced NRG-1 type I regulate remyelination after nerve injury [61,62,63,64]. NRG-1 also plays a role in the regulation of SC apoptosis during development. Axonal NRG-1 provides axonal size information to SCs, and decreased or excessive expression of neuregulin results in hypomyelination or hypermyelination, respectively [65,66]. SC proliferation, derived from both myelinated and unmyelinated axons, peaks 3–4 days after injury and then declines [24].

**Figure 4:**
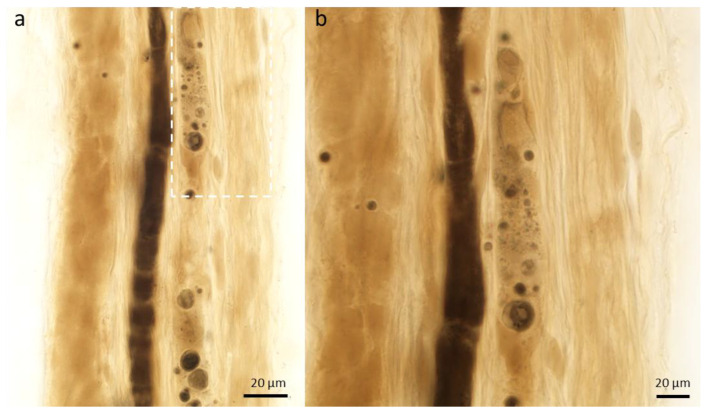
Axonopathy. Activity of macrophages and Schwann cells in the sural nerve of a patient with vasculitis. In panel (**a**), a macrophage outlined with a white dotted line is shown, and an enlarged view is provided in panel (**b**). (teased nerve fiber preparation; magnification: (**a**) 60× oil immersion; (**b**) 100× oil immersion).

Macrophages are important players in the process of WD [67,68,69]. After 3–4 days, the activation of both resident endoneurial macrophages and blood-derived macrophages occurs (Figure 4).

Cytokines and chemokines secreted by SCs are fundamental for macrophage chemoattraction and regulation, which lead to myelin clearance in WD. The most important are interleukin-1α, interleukin-1β (IL-1β), tumor necrosis factor α (TNF-α), monocyte chemoattractant protein 1 (MCP-1, or chemokine C-C motif ligand 2), macrophage inflammatory protein 1α, pancreatitis-associated protein III, leukemia inhibitory factor, and interleukin-6 [70]. In addition, macrophage migration depends on increased capillary permeability due to mast cell release of histamine and serotonin [11,71]. Some of the cytokines listed above, such as IL-1β, TNF-α, and MCP-1, also modulate the phagocytic ability of macrophages in injured nerves [70,72].

Together with SCs, macrophages play a critical role in removing myelin debris and promoting SC activation in axonopathies and contribute to the creation of a permissive environment for axonal regeneration in the distal stump by releasing several pro-regenerative factors, including cytokines and chemokines, growth factors, and extracellular matrix molecules [73,74].

**Figure 5:**
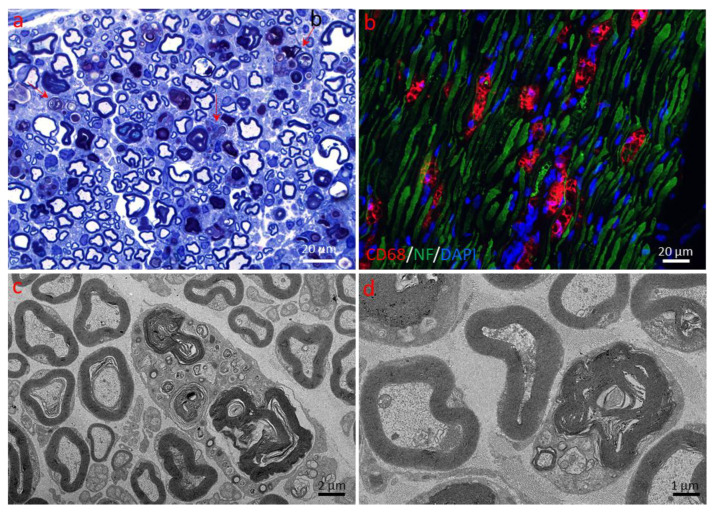
Axonopathy. Macrophage and Schwann cell activity in sciatic nerves of a 120-day-old mSOD1-G93A mouse. (**a**) Several macrophages (red arrows) are shown in the endoneurium in a 0.8 µm thick toluidine blue-stained Epon section (magnification: 100× oil immersion). (**b**) Immunohistochemical analysis of the mSOD1-G93A mouse sciatic nerve (green: neurofilament marker NF200; red: macrophage marker CD68; blue: nuclear marker DAPI) (40× magnification). (**c**,**d**) Electron micrographs of an activated macrophage (**c**) and Schwann cell (**d**), respectively (magnification: (**c**) 750×; (**d**) 1500×).

Even in diseases such as amyotrophic lateral sclerosis, macrophages play a key role, with macrophage infiltration in peripheral nerves beginning as early as the presymptomatic stages in mutant Cu/Zn superoxide dismutase 1 G93A (mSOD1)-transgenic mice, where distal axonopathy emerges in the initial phases (Figure 5) [75]. Macrophages expressing the C-C chemokine receptor type 2 seem to exert a protective role by contributing to the clearance of mSOD1 from peripheral nerves [75].

Collectively, these observations point to the fundamental role of the SC and macrophage secretome in regulating axonal degeneration and promoting axonal regeneration and nerve repair after injury [76].

#### 2.1.3. Regeneration of the Nerve Fiber

SCs and macrophages contribute to forming a microenvironment in the distal stump that supports axonal regeneration [71]. Denervated SCs undergo remarkable biochemical and morphological transformations and switch to the repair SC phenotype [51,58,59,77]. This topic has recently been reviewed by Jessen and Arthur-Farraj (2019) and Arthur-Farraj and Coleman (2021) [51,77]. Both SCs and macrophages exert their trophic functions and guidance roles in inducing and directing axonal regeneration by directly or indirectly driving the expression of several molecules with neurotrophic activity. To summarize this process, transcription factors, signaling pathways, and epigenetic changes have been described to promote axonal regeneration [78]. Some of them have been discussed in the previous paragraphs. Briefly, the transcription factor c-JUN acts as a central regulator of repair SCs through a constant and balanced interplay with many repair program genes, such as *sonic hedgehog*, *brain-derived neurotrophic factor*, and *glial-derived neurotrophic factor* [54,59,79]. Other neurotrophic factors are involved in the process of axonal regeneration: fibroblast growth factor 2, leukemia inhibitory factor [80], and insulin-like growth factor-1 (produced by SCs and macrophages) [81] and its receptor. Similarly, several cytokines (interleukins 6, 10, 12, and interferon γ); cell surface molecules (NCAM, L1, ninjurin 1, ninjurin 2) [82]; tenascin and N-cadherin, integrins that interact with type 2 laminin; and collagen types IV and VI contribute to the formation of the extracellular matrix in the context of regeneration [83,84]. In addition, there are other transcription factors involved in the process of SC-supported axonal regeneration, such as the POU domain transcription factor OCT-6, which is upregulated by SCs after injury and appears to repress c-JUN induction and delay axon regeneration [85]; the signal transducer and activator of transcription 3 (STAT3), which promotes the long-term survival of SCs after nerve injury and maintains the expression of c-JUN and other repair program genes [86]; the transcription factor Zinc Finger E-Box Binding Homeobox 2 (ZEB2); and two transcriptional activators of the Hippo pathway, yes-associated protein 1 (YAP) and tafazzin (TAZ) protein, which are required for SC remyelination after injury but not for the initial formation of repair SCs, myelin clearance, or c-JUN upregulation [51]. Several signaling pathways are activated in SCs soon after injury: the mammalian target of rapamycin (mTOR) signaling pathway, which is a component of the phosphatidylinositol 3-kinase cell survival pathway; the Notch signaling pathway, which plays an important role in controlling the rate of demyelination in injured nerves, although it remains unknown whether it regulates the formation and activity of repair SCs; and the Raf-MEK-ERK MAPK signaling pathway, which is important in regulating the expression of cytokines and chemokines by SCs and macrophages [77]. Finally, epigenetic factors, such as chromatin remodeling enzymes, non-coding ribosomal ribonucleic acid, and deoxyribonucleic acid methylation, regulate the SC injury phenotype and promote axonal regeneration [77,87]. Nerve injury induces the demethylation of the repressive histone mark H3K27 trimethylation at enhancers of several repair program genes. The complex interplay between epigenetic factors and the expression of SC injury genes, such as c-JUN, trophic and transcription factors, and neuregulins, was reviewed by Arthur-Farraj and Coleman in 2021 [51], and a detailed description of these mechanisms is beyond the scope of this paper.

At the morphological level, axons grow along the original basal lamina of the SC–axon unit and along the surface of SCs whose processes form the bands of Büngner. Histopathologically, it is difficult to distinguish reinnervated bands of Büngner from Schwann processes alone. In SC processes, light microscopy may reveal the presence of significant aggregates of ribosomes, increased SC cytoplasmic osmiophilia, and the prominence of cytoplasmic filaments. In axonal processes, MTs and neurofilaments can be identified instead [24]. The number of SCs is not critical in supporting axonal elongation, as the basal lamina remains continuous, confirming that extracellular matrix proteins play a key role in axon regeneration [88].

**Figure 6:**
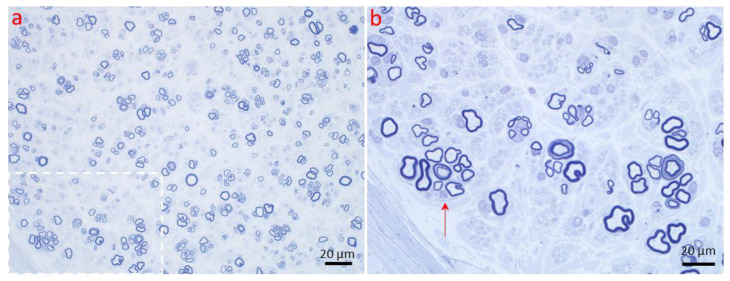
Axonopathy. Regeneration of the nerve fiber. (**a**,**b**) Clusters of small regenerating axons enwrapped by the original Schwann cell basal lamina can be seen by light microscopy. (**b**) Magnified view of the region marked in a to emphasize one of these axon clusters (red arrow). (1 µm thick toluidine blue-stained Epon section, magnification: (**a**) 40×; (**b**) 100× oil).

Within one day after transection, axonal regeneration begins in the stump proximal to the injury site from the most distal part of the damaged axon or from nearby nodes of Ranvier. A large number of regenerating sprouts from the nerve section can be seen after 5 days [24]. To sustain axon regeneration, intense protein synthesis is needed, which occurs mainly in the perikarya but also in the distal stump of regenerating axons. In particular, there is an increased expression of the cytoskeletal proteins peripherin and β-tubulin, as well as growth-associated protein 43 (GAP-43), which is a component of the growth cone [89]. Following this active protein production, axonal growth cones accumulate actin and tubulovesicular membrane elements. Clusters of small regenerating axons enwrapped by the original SC basal lamina can be seen by light microscopy (Figure 6).

In regenerating axons, the length of the myelinated internode is shorter than that of the original internode. In teased fiber preparations, regular, short, myelinated internodes are seen after axonal regeneration following axonal injury. Instead, after demyelination, the short internodes are intercalated with internodes of normal length (the original, undamaged internodes). Interestingly, the morphometric study of teased fibers confirms this morphological observation and may help us to understand the pathological mechanism underlying axonal degeneration in the individual axons. While the plot of internodal length versus axonal diameter in normal nerves shows a linear relationship, axons that have undergone degeneration and regeneration have a uniform internodal length (200–400 μm) despite their different diameters [24]. Occasionally, large clusters of frustrated regenerating axons give rise to a traumatic neuroma due to the accumulation of supernumerary axons [24].

A major problem in achieving anatomically and functionally efficient reinnervation of muscle and sensory targets depends on the many possible levels of misrouting of regenerating nerve fibers. More importantly, little is known about the mechanisms by which regenerating axons result in precise matching of the original axon with its SC. After nerve transection, even though neurotrophic factors are differentially expressed by myelinating SCs in the sensory and motor pathways and SCs previously associated with motor neurons express L2/HNK-1 only when contacted by motor axons [90], motor and sensory neurons often regenerate their axons into inappropriate channels, resulting in the reinnervation of functionally unrelated end organs.

**Figure 7:**
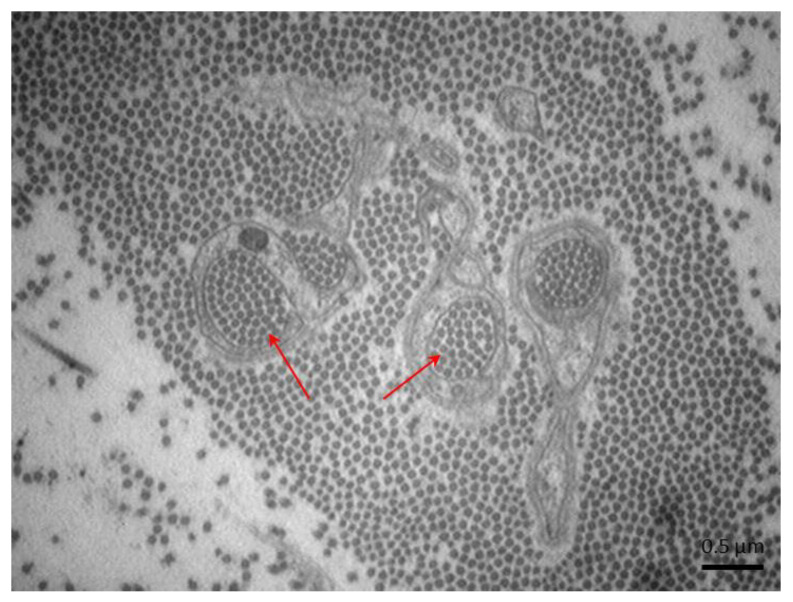
Collagen pockets (red arrows) surrounded by Schwann cell processes (electron microscopy, 4.400×).

As the neuropathic process becomes chronic and severe, few surviving axons remain along with scattered fibroblasts and a small number of SCs. The loss of unmyelinated axons results in the formation of “collagen pockets” in which longitudinal bundles of collagen are held in place of axons by SCs (Figure 7) [24]. This process can be deceptively unimpressive under light microscopy [24].

### 2.2. Axonal Atrophy

**Figure 8:**
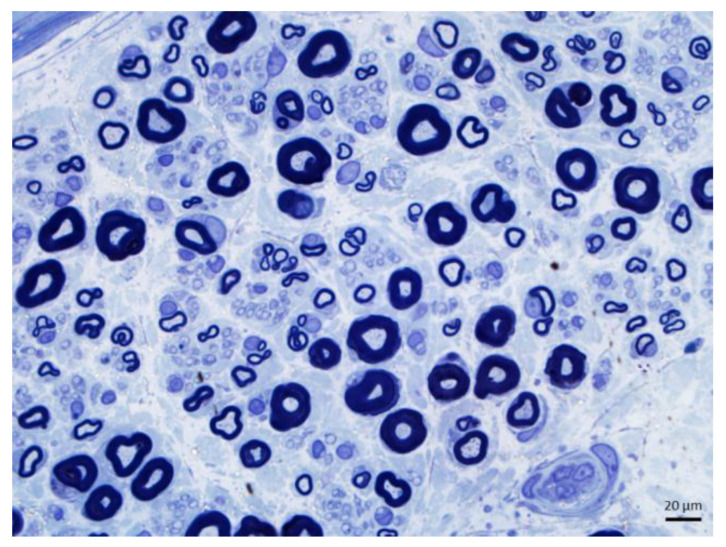
Axonopathy. Axonal atrophy. Several fibers exhibit a reduced axon diameter relative to the myelin sheath thickness (1 µm thick toluidine blue-stained Epon section, 100× oil immersion).

Axonal atrophy can be observed after permanent axotomy and in several types of neuropathies such as Charcot–Marie–Tooth (CMT) and related disorders, Friedreich’s ataxia, immune-mediated neuropathies later in the disease course, toxic neuropathies, and HIV- and HCV-associated neuropathies [48]. The g-ratio is calculated as axon diameter/(axon + myelin diameter) and is a complex evolving geometrical parameter that increases or decreases in function of the axon and myelin size. Morphologically and morphometrically, axonal atrophy is characterized by a reduction in the axon diameter and a consequent lowering of the g-ratio. As the axon undergoes atrophy, it often becomes smaller, appearing to be separated from its surrounding myelin sheath by lucent adaxonal SC cytoplasm; accordingly, the myelin appears wrinkled, with bubbles and loops [24] (Figure 8). Of note, the axon diameter increases during physiological development to generate larger caliber axons; if this growth is delayed, smaller-caliber axons accumulate, and the g-ratio decreases compared to normal conditions. This axonal maturation delay has been recently demonstrated in CMT1A neuropathy [91]. Therefore, a lower g-ratio may suggest either axonal atrophy or a failure in the physiological development of the largest myelinated fibers.

In the vicinity of the node of Ranvier, detachment between the axon and SCs is normal, and the axon appears smaller than in the internodal segment.

A molecular hallmark of axonal atrophy is the dephosphorylation of neurofilament light chain (NfL) proteins, resulting in increased NfL density and compaction [48]. Plasmatic levels of NfL are considered reliable fluid biomarkers for axonal degeneration and atrophy [92]. Interestingly, mutations in the genes encoding for NfL have been described in amyotrophic lateral sclerosis and some forms of axonal CMT, where axonal degeneration and atrophy are typical features. Elevated plasma levels of NfL have been found in several neurodegenerative diseases, including Alzheimer’s disease and Parkinson’s disease. The blood levels of NfL are higher in acute than in chronic peripheral nerve pathology. However, high levels of NfL have been observed in inflammatory demyelinating neuropathies, hereditary neuropathies such as CMT and transthyretin amyloidosis, and chemotherapy-induced neuropathies. The detection of high plasmatic levels of NfL may be predictive of ongoing axonal atrophy in the chronic progressive phase of neuropathy [93].

### 2.3. Axonal Dystrophy

Neuroaxonal dystrophy (NAD) is the hallmark of a group of rare, clinically and genetically heterogeneous neurodegenerative disorders in which distal axons in the CNS and PNS swell and accumulate various types of structured material (axonal spheroids) [94,95].

Inherited, primary, or endogenous NADs constitute a group of severe neurodegenerative diseases affecting not only humans [96,97,98,99] but also horses, cats, rats, and several breeds of dogs and mice [100,101,102,103,104,105,106,107,108,109,110,111]. The prototype of NAD in humans is infantile neuroaxonal dystrophy (INAD). INAD is a rare inherited neurological disorder that affects central and peripheral axons and is characterized by the progressive loss of vision, muscle control, and mental abilities. Mutations in the calcium-independent phospholipase A2 gene *PLA2G6* have been identified in most individuals with INAD. Pantothenate kinase-associated neurodegeneration, a form of neurodegeneration with brain iron accumulation, represents another variant of human NAD. This condition is characterized by axonal dystrophy and the formation of axonal spheroids within swollen axonal terminals, primarily in the basal ganglia, resulting from mutations in the *PANK2* gene, which encodes pantothenate kinase 2, a key enzyme in coenzyme A biosynthesis [94]. Patients typically present with extrapyramidal syndromes and cognitive decline.

The term “spheroid” does not describe the nature of the underlying axonal damage or distinguish between potential causative mechanisms. Possible pathogenetic mechanisms of axonal dystrophy include “synaptic dysplasia” (an abnormal outcome of normal cycles of synaptic degeneration and regeneration), frustrated axonal regeneration, or an imbalance of orthograde, retrograde, and turnaround axonal transport [112].

**Figure 9:**
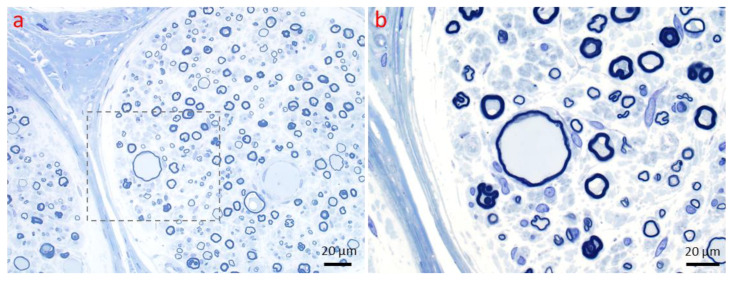
Axonopathy. Axonal dystrophy. (**a**,**b**) Giant axonal neuropathy. Many myelinated fibers appear distended and display attenuated myelin sheaths. (**b**) Magnified view of the region marked in a to emphasize one of these fibers. (1 μm thick toluidine blue-stained Epon sections; magnification: (**a**) 40×; (**b**) 100× oil immersion).

Axonal spheroids develop in all variants of endogenous NAD and are also observed in giant axonal neuropathy (Figure 9), a severe childhood-onset sensory–motor neuropathy characterized by CNS involvement and distinctive kinky hair, caused by mutations in the gene encoding gigaxonin [113]. Several neuropathologic conditions, including infectious, demyelinating, toxic, or traumatic injury, can also damage normal axons and produce spheroids, both in the CNS and PNS. However, electron microscopy shows that these “secondary” spheroids differ significantly from the spheroids found in primary axonopathies or endogenous NAD. For example, degenerated axons predominate in secondary axonopathies and consist of electron-dense bodies, whereas the spheroids in primary axonopathies contain organelles and structural elements that accumulate as a result of defects in axonal transport [114,115].

Although similar lesions are seen in various mammalian species with spontaneously occurring NAD, appropriate animal models to study the natural history, biochemical composition, and possible underlying mechanisms of spheroid formation are limited. In 2006, Bouley et al. described the neuropathologic features of a spontaneously occurring NAD mouse that closely resemble the ultrastructural changes seen in the axons of human patients with endogenous forms of NAD [116]. Neuropathological studies in another mutant model, the Gracile axonal dystrophy mouse, revealed inactivation of the ubiquitin carboxy-terminal hydrolase UCH-L1, a member of the ubiquitin–proteosome system, suggesting that the accumulation of organelles at the nerve terminal is due to their lack of degradation [117].

## 3. Myelinopathy

### 3.1. Primary Segmental Demyelination

In the PNS, axons and myelinating SCs form a unique symbiotic unit based on a finely tuned network of molecular signals and reciprocal interactions [118]. The importance of this complex interplay becomes apparent after injury or in diseases in which aspects of axo-glial interaction are disrupted. Molecular signals from the SC drive the distribution of sodium and potassium channels at the level of specific domains of the node of Ranvier (node and juxtaparanode), influence the phosphorylation of neurofilaments, and guide axonal growth by maintaining slow anterograde axonal transport [119,120,121].

Axons and SCs are interdependent for long-term survival and integrity; acquired or genetic defects in either cell type can cause a variety of acquired or inherited peripheral neuropathies [122].

In many inherited neuropathies, the term dysmyelination is more accurate than demyelination because the pathology involves abnormal myelin development rather than the destruction of previously normal myelin [123]. Nevertheless, hereditary dysmyelinating neuropathies are traditionally classified as demyelinating [124].

**Figure 10:**
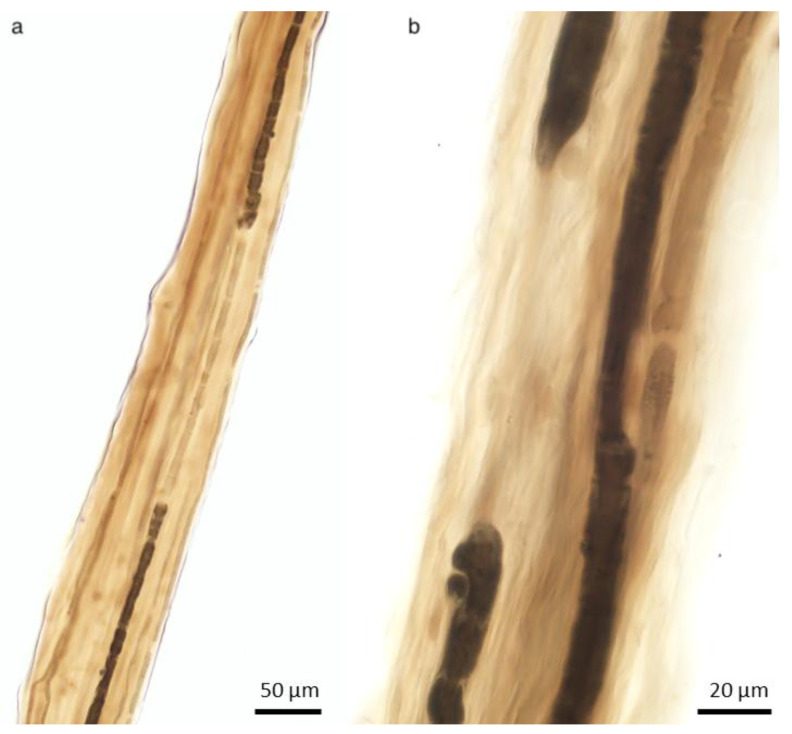
Myelinopathy. Segmental demyelination. Selective damage to the Schwann cell or the myelin itself results in myelin sheath degeneration and segmental demyelination, morphologically characterized by the loss of one or more internodes with relative preservation of axonal integrity. (teased nerve fiber preparation; magnification: (**a**) 20×; (**b**) 60× oil immersion).

Selective damage to the SC or the myelin itself results in degeneration of the myelin sheath and segmental demyelination, which is morphologically characterized by the loss of one or more internodes with the relative preservation of axonal integrity (Figure 10).

**Figure 11:**
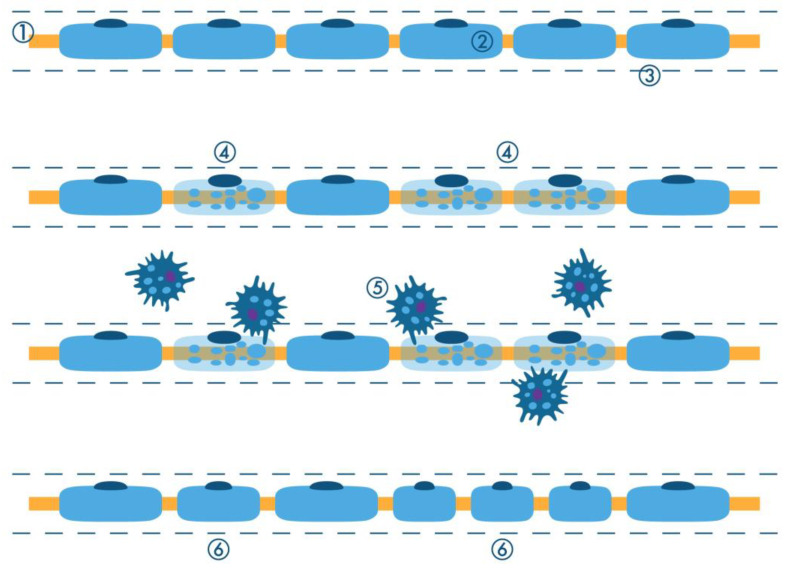
Scheme of segmental demyelination. 1 = axon; 2 = Schwann cell; 3 = basal lamina; 4 = myelinopathic injury; 5 = macrophages phagocytizing myelin debris; 6 = regeneration with shorter internodes.

Myelin damage begins with subtle vesicular degeneration followed by splitting of myelin lamellae, widening of the nodal gap, or abnormal folding of the myelin sheath [24]. If the original SC survives the insult, myelin debris accumulates in the SC cytoplasm and is engulfed by hematogenous and intrinsic macrophages, as occurs after axonal degeneration (Figure 11). Myelin debris is usually cleared rapidly, so the finding of macrophages containing this debris after several weeks suggests ongoing demyelination [24]. Macrophages may be the primary effector cells of active demyelination, as in acute or chronic immune-mediated polyradiculoneuropathies, in which macrophages strip otherwise normal-appearing myelin or in some experimental inherited neuropathies [125].

Segmental demyelination can be reproduced in vitro by co-culturing SCs and axons from transgenic rat models of inherited neuropathies [126], or by treating co-cultures with toxins such as cuprizone or the calcium ionophore A23187, which increases Ca^++^ leak into the SC cytoplasm, activates endogenous phospholipase A2, and causes demyelination [24].

The remyelination of individual internodes may occur after SC proliferation, typically resulting in the formation of multiple shorter intercalated internodes in place of the original internode. During the regeneration process, redundant SCs may undergo atrophy and decrease in number [48]. However, if cycles of demyelination occur prior to the elimination of redundant SCs, a cyclic process of demyelination/remyelination may be initiated, leading to the formation of hypertrophic “onion bulbs” [24].

**Figure 12:**
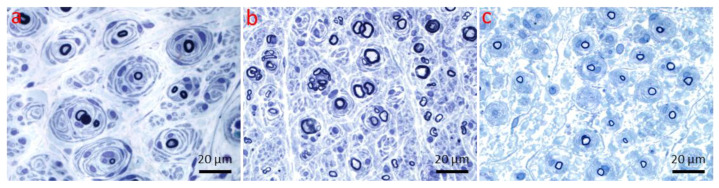
Myelinopathy. Onion bulbs. (**a**) Onion bulbs in a patient with CMT1A (1 μm thick toluidine blue-stained Epon section, 100× oil immersion). (**b**) Onion bulbs in a patient with anti-myelin-associated glycoprotein neuropathy (1 μm thick toluidine blue-stained Epon section, 100× oil immersion). (**c**) Onion bulbs in a patient with multifocal acquired demyelinating sensory and motor neuropathy (Lewis–Sumner syndrome) (1 μm thick toluidine blue-stained Epon section, 100× oil immersion).

Onion bulbs are typically observed in inherited neuropathies such as CMT1A, CMT1B, and Refsum’s disease, but also in chronic inflammatory demyelinating polyneuropathy (CIDP) and anti-myelin associated glycoprotein (MAG) neuropathy (Figure 12). Anti-MAG polyneuropathy, clinically characterized by prominent ataxia and neurogenic tremor, is associated with the widening of the space between the dense lines of the myelin sheath, caused by monoclonal IgM antibodies separating the intraperiod lines, primarily at the outermost myelin lamellae. Neurophysiologically, it is marked by a notable increase in distal motor latencies, disproportionately greater than the conduction velocity slowing observed in more proximal nerve segments. This imbalance is likely attributable to a length-dependent neuropathic process resulting from disrupted myelin–axon interaction, with a centripetal progression over time [124].

The presence of onion bulbs has traditionally been considered the result of repeated cycles of demyelination and remyelination, reflecting a process that has continued for several months or more. While this is true for inflammatory forms such as CIDP, it does not apply to inherited neuropathies. In CMT1A, onion bulbs appear to reflect abnormal SC differentiation that occurs during development [91,123,127].

Teased fiber analysis is particularly useful for detecting a demyelinating component in neuropathic processes, as it allows for the identification of segmental demyelination [3]. When the axonal diameter is plotted against the internodal length in teased fiber preparations, previously demyelinated and remyelinated nerves show an increased variation from node to node. This pattern is due to the replacement of the original myelin derived from a single SC with myelin produced by many proliferating SCs with smaller territories [24].

**Figure 13:**
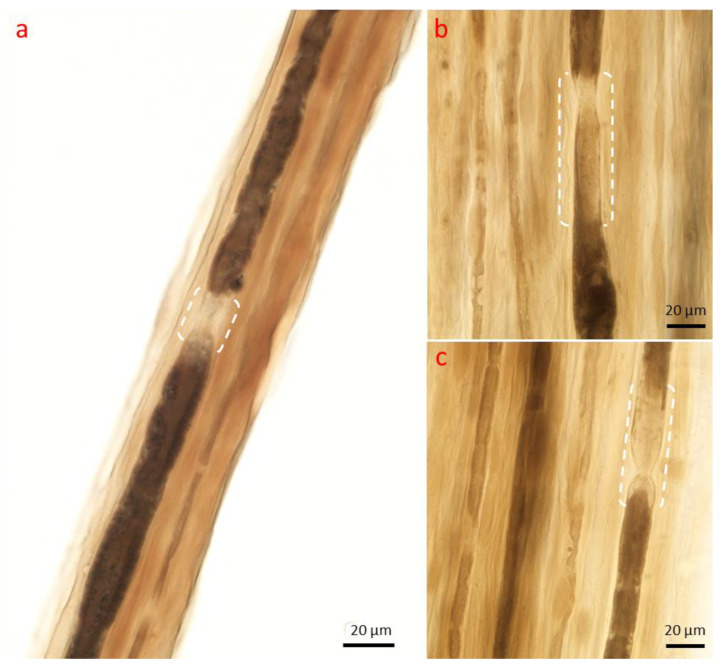
Paranodal demyelination. (**a**–**c**) Several instances of paranodal demyelination characterized by the typical widening of the nodes of Ranvier (indicated by dotted white parentheses). Teased nerve fiber preparation (magnification: (**a**) 60× oil immersion; (**b**,**c**) 100× oil immersion).

Additionally, teasing can reveal paranodal demyelination (Figure 13), characterized by widened nodes of Ranvier. Nodal widening may be observed without other signs of segmental demyelination in conditions such as autoimmune paranodopathies and acute motor axonal neuropathy (AMAN). Therefore, nodal widening should not be considered exclusive evidence of segmental demyelination [124].

In addition to sodium and potassium channels, the molecular organization at nodes, paranodes, and juxtaparanodes includes the expression of several different proteins such as neurofascins, contactin, contactin-associated proteins (Caspr), transient axonal glycoprotein 1, and ganglioside GM1 [128]. Neurofascin-186 (NF186), localized at the axolemma, is crucial for voltage-gated sodium (Nav) channel clustering via gliomedin interaction [128]. The paranodal junction consists of three major proteins: contactin-1 (CNTN1) and Caspr1 on the axonal side and neurofascin-155 (NF155) on the terminal myelin loops. The paranodal junction maintains adhesion between the terminal myelin loops and the axolemma, functioning as an electrical and molecular barrier to limit ion diffusion [128]. These molecules can be the specific target of immune-mediated attacks leading to so-called nodopathies and paranodopathies. In these neuropathies, segmental demyelination does not occur, and conduction block can result in axonal degeneration, confirming the importance of the interplay between axons and SCs [128,129]. Neuropathological analysis has aided in characterizing these disorders, which neurophysiologically resemble demyelinating neuropathies but are primarily defined by axonal degeneration. AMAN, a subtype of Guillain–Barré syndrome associated with IgG anti-GM1 antibodies, is characterized by conduction blocks and conduction slowing that initially mimic demyelination. However, unlike inflammatory demyelinating polyneuropathies, these abnormalities resolve without temporal dispersion. This phenomenon, termed reversible conduction failure (RCF), differs from the classical conduction block associated with demyelination due to its rapid resolution and the absence of slow components typical of remyelination [128,130,131]. Patients with AMAN and RCF frequently exhibit paranodal detachment, widening of the nodes of Ranvier, and axonal degeneration, supporting the concept of a pathological continuum in AMAN ranging from reversible axonal dysfunction to irreversible axonal degeneration [130,132]. The nodes of Ranvier, which possess the highest density of Nav channels, facilitate the regeneration and rapid propagation of action potentials. Gangliosides contribute to the integrity of neuron–glia interactions and ion channel clustering at the paranodes. The juxtaparanodes, adjacent to the paranodes beneath the myelin sheaths, are enriched in voltage-gated potassium (Kv) channels. In AMAN, anti-GM1 antibodies disrupt the paranodal structures, causing the mislocation of Kv channels. Complement activation by anti-GM1 antibodies leads to membrane attack complex (MAC) formation, which damages the nodal axolemma and disrupts nodal molecular complexes, including Nav channels. This autoimmune process induces paranodal detachment, nodal elongation, and conduction failure. The loss of terminal myelin loops results in current leakage, dissipating the driving current. Consequently, depolarization may become insufficient to activate the necessary Nav channels, leading to action potential failure [128,130]. Although nodal dysfunction and conduction failure may initially be reversible, persistent immune activation can lead to axoplasmic Na^+^ accumulation. Energy failure depletes ATP, impairing Na^+^/K^+^ pump function and causing membrane depolarization. This depolarization activates persistent Na^+^ channels, further increasing Na^+^ influx. If the immune response progresses, excessive axoplasmic Na^+^ levels trigger a reversal of the axolemmal Na^+^/Ca^++^ exchanger, leading to Ca^++^ accumulation. Mitochondria release additional Ca^++^ due to the intense Na^+^ influx, further exacerbating axonal damage [130]. Anti-GM1 IgG also activates the classical complement pathway, promoting MAC formation and allowing further Ca^++^ influx through membrane pores. Intracellular Ca^++^ accumulation activates calpain, leading to the proteolytic cleavage of neurofilaments, mitochondrial dysfunction, and Wallerian-like degeneration. Macrophages subsequently infiltrate the periaxonal space, scavenging injured axons [130]. This mechanistic framework explains the variable recovery observed in AMAN, which can range from rapid and complete resolution to prolonged dysfunction with poor prognosis, depending on the extent of RCF and axonal degeneration [130]. Multifocal motor neuropathy (MMN), characterized by IgM antibodies against GM1, is associated with persistent motor conduction blocks without temporal dispersion, along with mild demyelination and axonal degeneration. Although the mechanism is not fully understood, IgM anti-GM1 antibodies may bind at the node, activating complement and leading to MAC formation, which disrupts ion channels and paranodal structures, causing conduction blocks and eventually axonal degeneration [130]. Recently, nodo-paranodopathies associated with specific antibodies directed against CNTN1, Caspr1, or neurofascins, mainly of the IgG4 class, have been distinguished from CIDP [133]. These disorders exhibit conduction blocks without temporal dispersion and lack segmental demyelination on nerve biopsy. They can have an acute or subacute onset, peculiar clinical features, and are typically characterized by a lack of complement activation and a poor immunoglobulin response [130,134]. In paranodopathies, detachment of terminal myelin loops leads to nodal lengthening and widening of the periaxonal space, causing current leakage with dissipation of the driving current. Kv channels displaced from the juxtaparanode to the paranode induce hyperpolarization, delaying action potential regeneration and slowing conduction velocity. When the safety factor of nerve conduction drops below a critical threshold, conduction failure occurs [130]. The disruption of axo-glial interactions due to IgG4 antibodies further contributes to axonal degeneration, though the precise mechanism remains unidentified [130].

### 3.2. Dysmyelination

Demyelination involves damage to fully formed myelin sheaths, whereas dysmyelination refers to a defect in the development of myelin [123]. Although the CMT classification distinguishes between demyelinating and axonal forms, recent evidence suggests that many forms traditionally classified as demyelinating, including the most common, CMT1A, are actually characterized by dysmyelination. In support of this hypothesis, the nerve conduction velocity in CMT1A is uniformly reduced across all nerves from early childhood [135,136] and remains stable throughout life [137,138,139]. Instead, in cases of active demyelination, as in acute inflammatory demyelinating polyneuropathy, CIDP, or anti-MAG polyneuropathy, one would expect more variable conduction velocities and temporal dispersion, which are typically absent in CMT1A [124]. The slowing of the conduction velocity in CMT1A could be explained, at least in part, by a developmental abnormality in internode formation, with uniformly shorter internodes [140,141]. Altered myelin thickness may also contribute to conduction slowing. In the CMT1A rat model, large-caliber fibers are reduced and hypomyelinated, whereas small-caliber fibers are hypermyelinated [91]. Although axonal degeneration has traditionally been considered secondary to myelin abnormalities, CMT1A is likely characterized by defective PNS development involving both myelin and axonal maturation, with abnormal axo-glial interactions [91]. In contrast to nerve conduction velocity, the amplitude of compound muscle action potentials, which reflects axonal degeneration, correlates with disease severity [142]. Although onion bulbs have traditionally been considered the result of repeated cycles of demyelination and remyelination, in CMT1A, they are likely caused by abnormal SC differentiation [127,137], which may lead to an excess of SCs during development [143,144,145]. The maturation defect appears to occur during the early stages of development [91]. It has been shown that SCs exhibit insufficient lipid biosynthesis during development, and there is an alteration in the lipid composition of myelin [146,147], with an imbalance between short-/medium- and long-chain lipids [91]. This may also affect myelin permeability and ion channel distribution [91].

### 3.3. Secondary Demyelination

As previously reported, axons and SCs are connected by intimate crosstalk and exchange of molecular messages. Because myelin breakdown occurs after axonal injury, primary axonopathy can lead to secondary demyelination. Unlike segmental demyelination, in which foci of demyelination are randomly distributed along individual axons, secondary demyelination is clustered along selected axons distal to degenerative axonopathy changes. The pathomechanisms of secondary demyelination involve a disruption of the relationship between the SC and its ensheathed axon. The morphological results are axonal atrophy, myelin wrinkling, and paranodal and segmental demyelination [83,148]. Secondary demyelination typically occurs in uremic neuropathy, although it can also be observed in several other neuropathies such as diabetes, acute intermittent porphyria, Friedreich’s ataxia, giant axonal neuropathy, Tangier disease, thiamine deficiency, and toxic neuropathies [48].

### 3.4. Myelin Thickening and Changes in Myelin Folding

**Figure 14:**
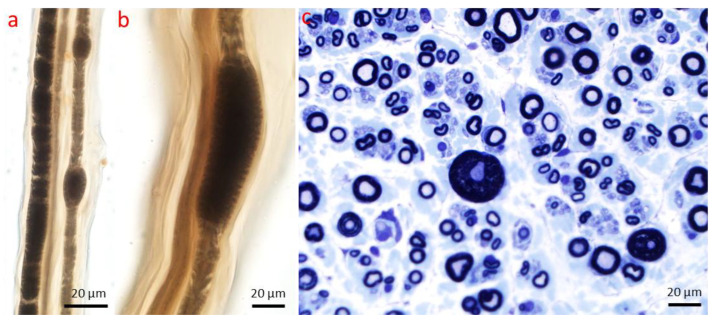
Myelinopathy. Tomacula. (**a**,**b**) Teased nerve fiber preparation shows focal thickenings of the myelin membrane (magnification: (**a**) 60× oil immersion; (**b**) 100× oil immersion). (**c**) Sural nerve biopsy displaying fibers with abnormally thick myelin sheath relative to axon diameter (magnification: 100× oil immersion).

Increased myelin thickness or redundant myelin sheath may sometimes be observed in inherited neuropathies and, more rarely, in a few acquired neuropathies. Focal thickening of the myelin sheath is a hallmark of hereditary neuropathy with increased liability to pressure palsies, also known as “tomacular” neuropathy, due to the typical formation of sausage-like focal thickening of the myelin sheath (“tomacula”, Figure 14). Due to their structural instability, tomacula make affected nerves more vulnerable to mechanical stress as repetitive movements or compression. They can result in focal conduction blocks, particularly at sites prone to compression, causing the characteristic recurrent episodes of focal weakness or sensory disturbances following minor traumas or pressure [149].

Other inherited neuropathies are characterized by redundant myelin in the form of myelin outfoldings, such as CMT1B, which is due to mutations in the gene encoding the MPZ protein, or CMT4B1, which is due to mutations in the myotubularin-related 2 (*MTMR2*) gene. Interestingly, a mouse model of CMT1B has recently been developed, exhibiting focal myelin thickenings identical to those observed in human CMT1B [150]. Teased fiber preparation is the most reliable histologic technique to diagnose hypermyelination in sural nerve biopsies.

## 4. Conclusions

Despite the progressive reduction in indications for nerve biopsy over the years, neuropathology remains a cornerstone in the diagnostic workup of specific peripheral nerve disorders. It continues to provide invaluable insights into the pathophysiological mechanisms underlying these diseases. Understanding the intricate cellular and molecular processes involved in axonopathies and myelinopathies is essential, particularly in light of recent advancements in therapeutic development. These breakthroughs highlight the need to unravel the complex interplay between axons and SCs, as well as the molecular mechanisms underlying peripheral nerve disorders, to identify novel therapeutic targets. By bridging the gap between clinical diagnostics and molecular biology, neuropathology holds a pivotal role in advancing our understanding of peripheral nerve disorders and driving the development of more effective, mechanism-based treatments.
